# Synchronous Occurrence of T-cell Lymphoblastic Lymphoma and High-Grade Glioma in a Pediatric Patient: A Case Report

**DOI:** 10.7759/cureus.84469

**Published:** 2025-05-20

**Authors:** Nikhil Thakur, Prabhjeet S Bathla, Sankalp Singh, Rajat Bahl, Jeenu Varghese

**Affiliations:** 1 Department of Radiation Oncology, Command Hospital Air Force, Bengaluru, IND; 2 Department of Radiation Oncology, Military Hospital, Kargil, IND; 3 Department of Hematology, Command Hospital Air Force, Bengaluru, IND; 4 Department of Pathology, Command Hospital Air Force, Bengaluru, IND

**Keywords:** cmmrd syndrome, constitutional mismatch repair deficiency, dual malignancy, glioblastoma multiforme, t-cell lymphoblastic lymphoma

## Abstract

T-lymphoblastic lymphoma (T-LBL) is a malignant transformation of lymphoid progenitor cells of T-cell origin. Gliomas are tumors arising from glial cells in the brain and spinal cord. The synchronous occurrence of these malignancies is rare. This case report presents a rare and challenging clinical scenario involving a seven-year-old male child diagnosed with T-LBL and a synchronous central nervous system (CNS) high-grade glioma (HGG). He received multiagent chemotherapy for T-LBL, underwent surgery, along with adjuvant radiation therapy for HGG. However, due to the progression of the intracranial disease, he succumbed to this illness. Secondary malignancies, especially CNS tumors, are well recognized in survivors of acute lymphoblastic leukemia who have undergone prior prophylactic cranial irradiation. The case highlights the rarity of co-occurrence of T-LBL and CNS HGG in a patient without prior cranial irradiation.

## Introduction

Acute lymphoblastic leukemia/lymphoblastic lymphoma (ALL/LBL) is a malignant transformation and proliferation of lymphoid progenitor cells in the bone marrow, blood, and extramedullary sites. It is classified into B-cell ALL, T-cell ALL, and natural killer cell ALL. World Health Organization (WHO) Classification of Tumors of Hematopoietic and Lymphoid Tissues in their Fifth Edition classifies T-cell ALL/T-lymphoblastic lymphoma (T-LBL) as precursor lymphoid neoplasm originating from T-cell lineage, comprising T-lymphoblastic leukemia/LBL NOS and early T-precursor lymphoblastic leukemia/LBL [1].

The central nervous system (CNS) involvement in ALL is seen in 3%-5% of patients at the time of initial diagnosis and 30%-40% at the time of relapse [2]. The CNS involvement is diagnosed by microscopic examination of cerebrospinal fluid (CSF) for blast cells and WBCs. The CNS status is then classified into CNS1, 2, or 3 depending on the presence of blast cells and the number of WBCs [3]. Multiple risk factors for CNS relapse have been defined, such as a T-cell immunophenotype, presence of BCR-ABL1, KMT2A (mixed-lineage leukemia) rearrangements, TCF3-PBX1 cytogenetic abnormalities, and hyperleukocytosis [2]. The treatment strategy is based on intensive multidrug chemotherapy, including CNS prophylaxis, with or without mediastinal radiation therapy. CNS-directed therapies in ALL consist of high-dose systemic therapy combined with intrathecal (IT) therapy or radiotherapy. The agents with significant CNS activity include IT and systemic glucocorticoids, methotrexate, cytarabine, and asparaginase [4].

Gliomas are the most common primary CNS neoplasms in children and adolescents and are thought to arise from their glial progenitors or stem cells. These may be roughly segregated into low-grade gliomas (WHO grade I or II; including most mixed glial-neuronal tumors) and high-grade gliomas (HGGs; WHO grade III or IV). The Fifth Edition of WHO Classification of Tumors of the CNS divided pediatric gliomas into pediatric type diffuse low-grade gliomas (consisting of diffuse astrocytoma, MYB-or MYBL-1 altered; angiocentric glioma; polymorphous low-grade neuroepithelial tumor of the young; diffuse low-grade glioma, and mitogen-activated protein kinase pathway altered) and pediatric type diffuse HGGs (consisting of diffuse midline glioma, H3 K26-altered; diffuse hemispheric glioma, H3 G34-mutant; diffuse pediatric type HGG, H3 wild type and isocitrate dehydrogenase, IDH, wild type; and infant type hemispheric glioma) [5]. They may occur anywhere in the CNS, but most often arise in the cerebral hemispheres or midline structures of the brain, where the pons and thalamus are more commonly affected than the cerebellum or spine. Some HGGs occur in patients with cancer predisposition syndromes such as constitutional mismatch repair deficiency (CMMRD), Li-Fraumeni’s syndrome, or neurofibromatosis type 1 (NF1), but the majority present as sporadic tumors with unknown etiology [6]. The synchronous occurrence of ALL and HGG is rare. Here, we report a case of a seven-year-old child with dual malignancy, T-cell ALL, and HGG.

## Case presentation

A seven-year-old male child presented with complaints of a prolonged cough with features of superior vena cava obstruction syndrome. He was evaluated with a chest X-ray suggestive of a large mediastinal mass. Fluorodeoxyglucose positron-emission computer tomography (FDG PET CT) scan revealed a metabolically active anterior mediastinal mass measuring 8 x 9 x 14.4 cm, with no evidence of any abnormal FDG activity elsewhere in the body. An image-guided Tru-Cut biopsy from the mediastinal mass showed a monomorphic population of small to intermediate lymphoid cells with scant cytoplasm, irregular nuclear membrane, and hyperchromatic nuclei, with areas of fibrosis and necrosis. Immunohistochemistry (IHC) revealed CD3 positive, Tdt positive, CD10 positive, Ki 67%-90%, CD20 negative, CD79a weak positive, and pancytokeratin negative. He was diagnosed with T-LBL. Bone marrow biopsy showed normal cellular marrow, with no atypical or malignant cells seen. He was started on an induction regimen (multiagent chemotherapy as per Children's Oncology Group protocol AALL0434, consisting of IT cytarabine, vincristine, prednisolone, daunorubicin, pegaspargase, and IT- methotrexate). He developed chemotherapy-induced severe cardiac and hepatotoxicity during phase II induction therapy, leading to an interruption in treatment. He was subsequently treated with a reduced dose as per protocol. He became symptomatic with headache and vomiting after completion of the phase II induction regimen. Contrast-enhanced magnetic resonance imaging (CEMRI) of the brain and spine showed an ill-defined intra-axial mass lesion measuring 30 (anteroposterior) × 25 (transverse) × 38 craniocaudal (CC) mm involving cortical and subcortical region of right parietal lobe, heterogeneously isointense to gray matter on T1-weighted images and heterogeneously hyperintense on T2-weighted and FLAIR images with restriction on diffusion weighted images and post-contrast enhancement and perilesional edema. Magnetic resonance spectrometry showed a choline peak. FDG PET CT scan showed the lesion in the right parietal lobe to be heterogeneously FDG avid measuring 2.4 x 2.8 x 4.1 cm (standardized uptake value - maximum, SUV_max_, 10.9), FDG avid conglomerate lymph nodal mass in the superior mediastinum measuring 4.7 x 7.8 x 6.7 cm (SUV_max_ 10.4) encasing and compressing superior vena cava, FDG avid conglomerate lymph nodal mass in anterior mediastinum measuring 1.4 x 3.1 x 2 cm, and mildly FDG avid (SUV_max_ 3.9) ill-defined soft tissue thickening epicentered in prevertebral and left para vertebral region along with dorsal vertebrae (DV) 6 to DV 10 with craniocaudal extent of 5.8 cm. CSF analysis did not detect any malignant cells. The differential diagnosis included a secondary CNS deposit from lymphoma, primary CNS lymphoma, and primary glioma.

**Figure 1:**
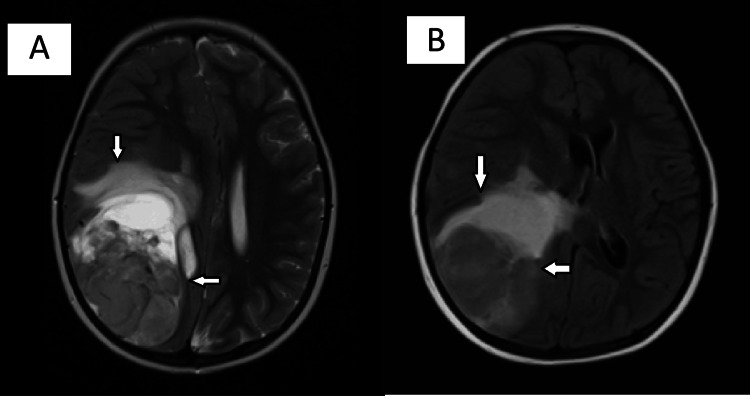
Contrast-enhanced MRI of the brain. (A) T2 contrast axial sections showing heterogeneously hyperintense intra-axial mass lesion measuring 70 x 49 x 60 mm, featuring a cystic area on its anteromedial aspect associated with perilesional edema. (B) T2 FLAIR axial sections showing heterogeneously hyperintense intra-axial lesion with cystic area MRI: magnetic resonance imaging; FLAIR: fluid-attenuated inversion recovery

Based on clinicoradiological findings, the patient was diagnosed as a case of ALL with CNS involvement and continued treatment with multiagent chemotherapy. However, the patient symptomatically worsened over a period of three months. CEMRI brain showed an increase in the size of the brain lesion measuring 70 x 49 x 60 mm, with an increase in perilesional edema and midline shift of 12 mm (Figure 1).

**Figure 2:**
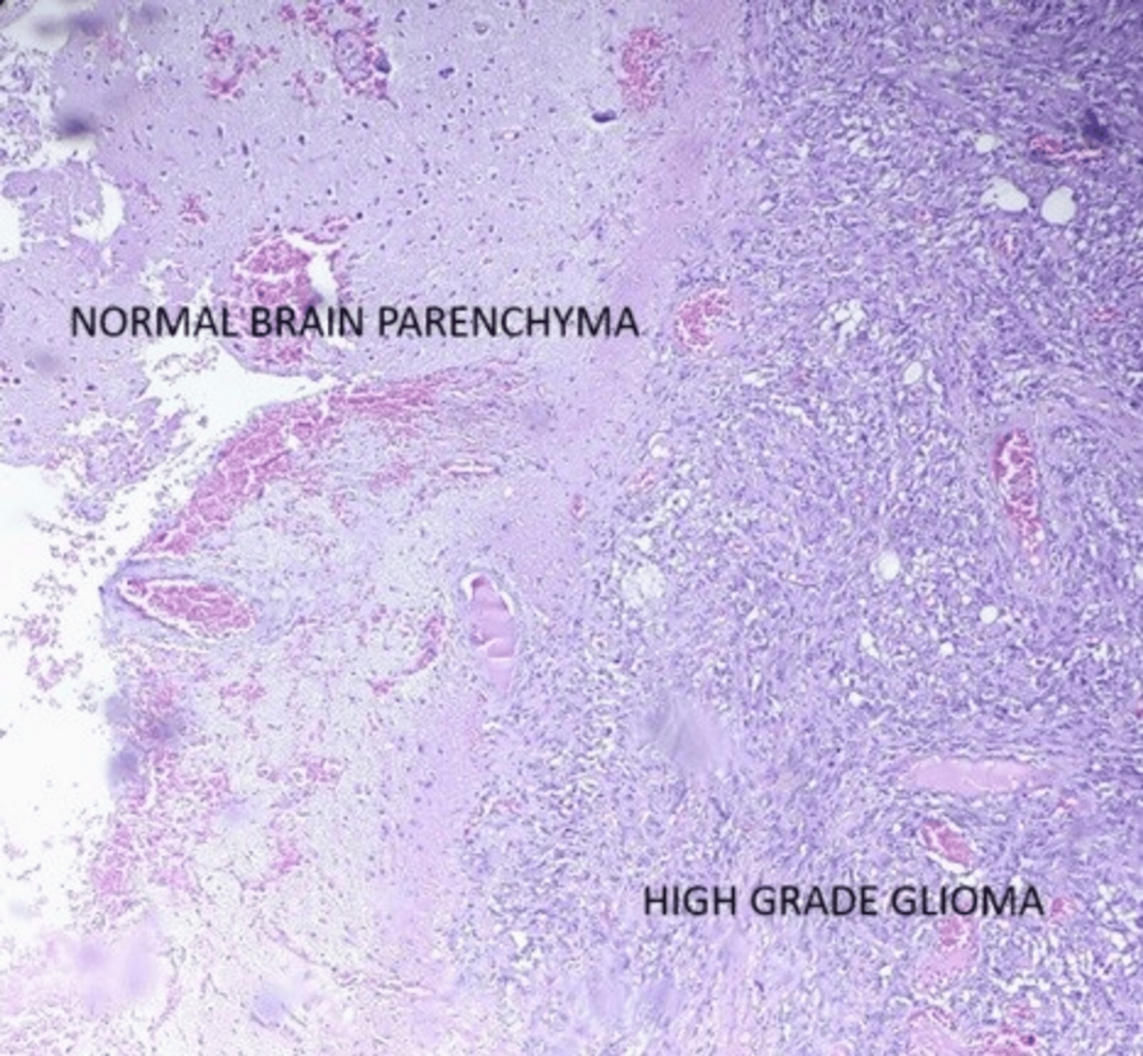
H&E 100× magnification showing well defined tumor demarcation from normal brain parenchyma H&E: hematoxylin and eosin

Noncontrast computed tomography of the chest showed a significant reduction in the size of the mediastinal lymph nodal mass and a few enlarged lymph nodes in the prevascular and right upper paratracheal region, measuring approximately 12 mm. After evaluation, the clinicoradiological assessment showed a primary mediastinal lesion responding well to treatment. However, the lesion in the brain continued to grow in size. The differential response to chemotherapy raised suspicion of a different pathology in the brain, and the patient was suspected to harbor a second primary brain pathology. He underwent decompressive craniotomy and maximal safe excision of the tumor. The histopathological examination of the resected tissue showed tumor infiltrating diffusely in sheets with a high N:C ratio, hyperchromatic nuclei, and scant cytoplasm with large areas of necrosis, suggestive of high-grade malignancy (Figure 2).

Immunohistochemistry revealed the tumor cells to be vimentin positive, glial fibrillary acidic protein positive, Olig2 positive, IDH1-R132H negative, ATRX retained, P53 positive, MIB1 labeling of 70%-80%, synaptophysin positive in undifferentiated cells, epithelial membrane antigen negative, L1CAM negative, leukocyte common antigen negative, BCOR negative, MSH2 retained, MSH6 retained, and MLH1 retained, and PMS2 showed loss of nuclear expression in tumor cells and endothelium. He was diagnosed as a case of glioblastoma (GBM) with primitive neuronal component, NOS, CNS grade IV.

At four weeks postoperatively, CEMRI brain showed a residual tumor with hyperintensities in the right parietal lobe and a small intradural nodule of 2 mm at LV1. The case was discussed in a multidisciplinary tumor board. Considering that the patient was already on a multiagent chemotherapy protocol for LBL, it was decided to proceed with adjuvant radiation to the brain only. Concurrent and adjuvant temozolomide were omitted from the treatment plan. He received adjuvant treatment with focal irradiation to a dose of 59.4 Gy in 33 fractions to the planning target volume as defined by preoperative T1 contrast and postoperative FLAIR/T2 images over a period of 61/2 weeks by intensity-modulated radiation therapy technique on a Tomotherapy Radixact (Accuray Incorporated, Madison, WI). Three months after radiotherapy, CEMRI of the brain revealed a mild reduction in the size of the residual lesion measuring 2.0 x 2.8 x 2.6 cm, a notable reduction in perilesional edema and mass effect. He completed maintenance chemotherapy with vincristine, prednisolone, and intrathecal methotrexate, and LBL was in remission. At 12 months after radiotherapy, the patient was admitted with altered sensorium and recurrent seizures, and succumbed to his illness due to the progression of intracranial disease.

## Discussion

The synchronous occurrence of T-LBL and HGG presents a complex and rare clinical scenario that warrants thorough discussion in a multidisciplinary forum. This unique combination of malignancies poses significant challenges in terms of diagnosis, treatment, and prognosis.

The development of secondary malignancies is well recognized among the survivors of hematological malignancy, and the entity of tumors comprises predominantly acute myeloid leukemia, myelodysplastic syndrome, or CNS tumors [7,8].

Neglia et al. reported that the cumulative risk of secondary neoplasm ranges from 1.7% to 3.3% after 10-15 years of follow-up. The study included 9,720 patients who underwent treatment for ALL. Among these patients, 43 patients were diagnosed with second neoplasm, of which 24 were CNS tumors consisting of HGG (14 patients), primitive neuroectodermal tumor (three patients), medulloblastoma (one patient), meningioma (two patients), brain stem glioma (one patient), low-grade glioma (two patients), and ependymoma (one patient). All the CNS neoplasms occurred in the children who had previously undergone cranial irradiation as part of ALL treatment [9]. Another study showed a cumulative incidence for secondary brain tumors of 1.39% at 20 years. However, in this study, there were also no instances of brain tumors in patients who did not receive cranial irradiation as part of their ALL therapy [10].

The peculiarity in our case is the diagnosis of a synchronous CNS neoplasm (HGG) in a patient of T-LBL who has not received any prior CNS irradiation. The diagnosis of T-LBL in our case is based on the biopsy from the mediastinal mass with extensive IHC study suggestive of T-cell lineage, bone marrow biopsy suggestive of normal cellular bone marrow, and imaging showing a mediastinal mass. The diagnosis of the GBM is based on histopathological and IHC assessment of the resected specimen.

The etiologies of brain tumors in most cases are unknown; only a fraction of brain tumors can be linked to genetic or environmental factors. However, with the advances in gene profiling and molecular testing, several predisposing syndromes have been identified in patients with CNS malignancies. These syndromes include NF1 and NF2, schwannomatosis, rhabdoid tumor predisposition syndrome, Gorlin, tuberous sclerosis, Von Hippel-Lindau, Li-Fraumeni, Turcot, and mismatch repair (MMR) deficiency syndrome [11,12].

We presented a case of a seven-year-old male child who developed synchronous T-LBL and GBM multiforme WHO grade IV, IDH wild type. The IHC revealed a loss of nuclear expression of PMS2, suggesting an MMR deficiency. The literature describes multiple syndromes associated with MMR gene defect/deficiency, including Lynch syndrome, CMMRD, Bi-allelic somatic mutations in suspected Lynch syndrome, familial colorectal cancer type X, and clinical lynch syndrome. The function of the MMR gene is to maintain genomic stability, and dysfunction can lead to alteration in the sequence number of microsatellites, defined as microsatellite instability (MSI). Lynch syndrome is caused by pathogenic germline variants in any of the four DNA MMR genes (MLH1, MSH2, MSH6, or PMS2) or by an EPCAM deletion. CMMRD is a biallelic mutation in the DNA MMR gene [13]. Lynch syndrome presents with malignancies in the fifth or sixth decade of life, while CMMRD develops cancers early in childhood in the first decade of life. The diagnosis of Lynch syndrome is based on revised Bethesda criteria and Amsterdam II criteria [14]. The diagnosis of CMMRD is based on the three-point scoring system of the European Consortium C4CMMRD [15]. In patients who are suspected to have CMMRD, the biopsy specimen can be tested by IHC for mismatch protein or MSI analysis. The final diagnosis is based on the demonstration of biallelic mutation of the MMR gene by sequencing or ligation-dependent probe amplification. CMMRD syndrome is associated with hematological malignancies, CNS tumors, LS-associated carcinomas, and premalignant and non-malignant lesions. Among the hematological malignancies in CMMRD, the most common is non-Hodgkin lymphoma of T-cell origin. HGGs constitute the most common CNS tumors in CMMRD. The most common gene affected in CMMRD is PMS2, unlike Lynch syndrome [15]. Syndromes such as CMMRD are extremely rare, with an incidence of one in a million patients [16].

The clinicopathological findings in our patient share multiple similarities with CMMRD syndrome, such as presentation in the first decade of life with T-LBL along with high-grade CNS glioma, with tumor cells showing loss of PMS2 expression.

## Conclusions

We present a rare co-occurrence of HGG in a patient of T-LBL. The synchronous occurrence of brain tumors and hematological malignancies has been defined in multiple syndromes. The clinical scenario presented in the report with a child T-LBL and HGG with loss of PMS2 suggests the patient is affected with CMMRD. We suggest a molecular evaluation of the patient for CMMRD and genetic counseling for further management. Such rare presentations prompt clinicians to undertake the challenges in managing dual malignancies in pediatric patients, urging continued exploration of underlying molecular mechanisms and treatment modalities. This study also highlights the importance of tissue sampling from every newly detected site of disease to confirm secondary deposits, as it can significantly change management. The report also reiterates the importance of a high index of suspicion for a second primary if there is a differential response to chemotherapy at two different sites of disease in the body. Our reported case scenario is rare because CNS malignancy has occurred in a pediatric patient with hematological malignancy synchronously and without exposure to brain radiation.
